# Z variant heterozygosity in alpha-1 antitrypsin deficiency: disease risk and treatment implications

**DOI:** 10.1186/s13023-026-04283-9

**Published:** 2026-04-08

**Authors:** Craig P. Hersh, Jeffrey H. Teckman, Pavel Strnad, Aaron Hakim, Tomás P. Carroll, Ian P. Hall, Auyon J. Ghosh, Igor Barjaktarevic, Noel G. McElvaney, Joseph E. Kaserman, David A. Lomas, Charlie Strange, M. Bradley Drummond, Stephen Rennard, Kathi E. Hanna, Virginia C. Clark, Monica P. Goldklang, Peg Iverson, Andrew A. Wilson

**Affiliations:** 1 Channing Division of Network Medicine, Brigham and Women’s Hospital, Boston, MA USA; 2 Division of Pulmonary and Critical Care Medicine, Department of Medicine, Brigham and Women’s Hospital, Boston, MA USA; 3 Harvard Medical School, Boston, MA USA; 4 Pediatrics and Biochemistry, Saint Louis University, Cardinal Glennon Children’s Medical Center, St Louis, MO USA; 5 Medical Clinic III, Gastroenterology, Metabolic Diseases and Intensive Care, University Hospital RWTH Aachen, Health Care Provider of the European Reference Network on Rare Liver Disorders (ERN RARE LIVER), Aachen, Germany; 6 Division of Gastroenterology and Hepatology, Beth Israel Deaconess Medical Center, Boston, MA USA; 7 Irish Center for Genetic Lung Disease, Department of Medicine, Royal College of Surgeons in Ireland, Dublin, Ireland; 8 School of Medicine, University of Nottingham, NIHR Nottingham BRC, NUH NHS Trust, Nottingham, UK; 9 Division of Pulmonary, Critical Care, and Sleep Medicine, SUNY Upstate Medical University, Syracuse, NY USA; 10 University of California Los Angeles David Geffen School of Medicine, Los Angeles, CA USA; 11 Center for Regenerative Medicine (CReM) of Boston University and Boston Medical Center, Boston, MA USA; 12 The Pulmonary Center and Department of Medicine, Boston University School of Medicine, Boston, MA USA; 13 UCL Respiratory, University College London, London, UK; 14 Institute of Structural and Molecular Biology, University College London, London, UK; 15 Division of Pulmonary, Critical Care, Allergy and Sleep Medicine, Medical University of South Carolina, Charleston, SC USA; 16 Division of Pulmonary Diseases and Critical Care Medicine, Department of Medicine, University of North Carolina, Chapel Hill, NC USA; 17 Department of Internal Medicine, University of Nebraska Medical Center, Omaha, NE USA; 18 Science and Health Writer/Editor, Seattle, USA; 19 Division of Gastroenterology, Hepatology, and Nutrition, University of Florida, Gainesville, FL USA; 20 Departments of Medicine and Anesthesiology, Columbia University, New York, NY USA; 21 Alpha-1 Foundation, Coral Gables, USA

**Keywords:** Alpha-1 antitrypsin deficiency, Alpha-1 antitrypsin, Rare disease, Heterozygote, Augmentation therapy, COPD, Chronic liver disease, Carrier state

## Abstract

**Background:**

Individuals heterozygous for alpha-1 antitrypsin deficiency (AATD) have one copy of the normal “M” allele and one copy of an abnormal allele (“Z”, “S”, or another variant) in the *SERPINA1* gene. Historically, evidence has been lacking to support the concept that heterozygotes are at increased risk for liver and/or lung complications compared to individuals homozygous for the M allele. However, growing evidence suggests that inheritance of a single Z allele increases the risk of disease in some individuals. The Alpha-1 Foundation convened a workshop on October 27, 2023, in Bethesda, Maryland that included stakeholders from the research, pharmaceutical, and patient communities. The focus of the meeting was to describe and assess what is known about the relative risks of liver and/or lung disease for heterozygotes and to identify future avenues of research into disease mechanisms and clinical phenotypes in MZ heterozygotes.

**Results:**

Population-based and family studies of individuals heterozygous for the AATD Z risk allele demonstrate that a proportion of these individuals have relatively higher risk for lung or liver disease than do individuals harboring no variants in *SERPINA1*. Included are studies identifying increased rates of chronic obstructive pulmonary disease (COPD) among MZ and SZ smokers compared to MM individuals with similar histories of smoke exposure. Evidence collected from human macrophages, cellular and mouse models of AATD, and explanted tissue from AATD patients provides clues to the disease mechanisms associated with Z heterozygosity. Early-stage research is focused on identifying the proportion of MZ individuals who might be at increased risk of disease and determining whether treatment strategies in current use or under investigation for ZZ patients might be appropriate and beneficial for Z heterozygotes. Participants discussed clinical trials design and infrastructure needed to study the AATD heterozygous population.

**Conclusion:**

Evidence at this time suggests modest increased risk among Z heterozygotes as a result of carrying a single Z gene. Associated risk of this population to develop either liver or lung disease is modified by genetic and environmental factors. Focused clinical trials are needed before using treatments beneficial for homozygous Z individuals in this population.

**Clinical trial number:**

Not applicable.

## Introduction

Alpha-1 antitrypsin deficiency (Alpha-1, or AATD) is an autosomal codominant genetic condition that can result in serious lung disease (emphysema and bronchiectasis) in adults and/or liver disease in infants and middle-aged adults. AATD most commonly results from a single base pair mutation in the *SERPINA1* gene (encoding AAT) that causes misfolding and associated reductions in circulating levels of AAT in the blood. AAT is a glycoprotein, primarily produced by the liver. Its primary known function is to protect the lungs from unopposed protease activity and proteolytic degradation by inhibiting neutrophil elastase, particularly in the context of inflammation caused by infection or inhaled irritants such as tobacco smoke. In individuals harboring two copies of the most common mutation associated with AATD, termed the “Z” allele (referred to as ZZ or Pi*ZZ), low levels of AAT in the blood occur because misfolded Z AAT protein accumulates intracellularly in liver hepatocytes and is not secreted in normal amounts. This intracellular accumulation of misfolded protein then leads to downstream liver disease in some patients.

Augmentation therapy with exogenous AAT is the only specific therapy for lung disease resulting from AATD. A spectrum of new therapies aimed at ameliorating lung disease, liver disease, or both is currently in development and includes neutrophil elastase inhibitors, small molecules to correct AAT misfolding, and recombinant versions of AAT as well as treatments targeting the gene or mRNA to either reduce expression of the abnormal form or induce expression of the normal form of the protein.

Approximately 100,000 people in the United States are living with severe AATD and similar numbers have been estimated to be affected in Europe. Worldwide, the number of people with severe AATD is 1 in every 1,500 to 3,500 people of European ancestry [1]. People identified with severe AATD typically have inherited two abnormal Z alleles. However, a number of gene variants exist in the population with a subset resulting in low AAT levels or altered function. The “S” allele is another common abnormal form of the gene, although less pathogenic than the Z allele. The normal allele is designated as “M.” Individuals with two M alleles are referred to as MM, homozygous for the normal *SERPINA1* allele. Gene combinations resulting in moderate deficiency can include SZ and MZ, although people with these combinations are less likely to develop lung or liver problems than those with two Z genes.

AAT heterozygotes have one copy of the normal M allele and one copy of an abnormal allele (Z, S, or another variant). Historically, evidence has been lacking to demonstrate that the Z heterozygous state confers increased risk for either liver or lung disease relative to the M homozygous (normal) state. This lack of data and the associated uncertainty regarding potential risk has resulted in uncertainty for both patients and practitioners and led to heterogeneity in treatment approaches. As a result, questions have persisted about the potential for lung and/or liver disease risk among heterozygotes.

In 2017, the Alpha-1 Foundation and its partners convened a workshop to assess what was known about the heterozygous state and identify future lines of research into disease mechanisms and clinical phenotypes in MZ heterozygotes. That workshop concluded with the development of a series of research questions to pursue regarding the clinical implications of MZ status, and possible approaches for answering them [2].

Understanding of the risks of Z heterozygosity has evolved in the intervening years. For example, SZ individuals were commonly treated with augmentation therapy before more recent evidence further characterized the risk associated with this genotype. Similarly, recent data have helped to quantify the relative risk of MZs to develop cirrhotic liver disease. Because of the implications for the enormous number of individuals around the world who harbor a single Z gene, it is incumbent to identify the relative risk for disease of this population and determine whether treatments beneficial for ZZ individuals might benefit this group as well.

In October 2023, the Alpha-1 Foundation convened its second workshop on this topic to re-examine the relative risk that Z heterozygotes have for injury to either the lung or liver in the context of more recent data. Speakers addressed the relative risks of lung and liver disease among heterozygotes, disease mechanisms in this population, and treatment options and research strategies. This is a summary of the presentations and discussions of that day.

## Session I: Relative risk of liver disease among Z heterozygotes

*Liver Disease Risk Among Z Heterozygotes with Other Known Liver Disease Risk Factors*

Pavel Strnad, MD, University Hospital Aachen

The frequency of the ZZ genotype varies worldwide and on average appears in roughly 1 in 2,000 individuals in Europe. In contrast, the frequency of the SZ genotype is approximately 1 in 500 individuals and the MZ genotype can be as high as 1 in 30. As such, the risk of disease in the SZ and MZ populations could be significant in population terms and screening could identify those at relatively greater risk for liver disease [3].

The Z variant produces a loss of function phenotype in the lung and a gain of function phenotype in the liver due to an excess of misfolded AAT. Increased susceptibility to liver fibrosis has been clearly confirmed even in the MZ population, as evidenced by the roughly 10% of total liver transplant recipients of European descent with an MZ genotype [4]. The challenge lies in identifying MZ individuals who are likely to be asymptomatic or have only mild symptoms despite having significant liver fibrosis.

The UK Biobank proved to be a great resource since it identified 17,181 MZs through systematic genotyping. Although there has been no detailed assessment of liver function in this population, baseline phenotype assessments have been used to evaluate liver enzyme values across genotypes. In addition, the available ICD codes revealed a markedly increased occurrence of liver fibrosis/cirrhosis and primary liver cancer in individuals with the ZZ genotype [3]. Subjects with the MZ genotype had slightly elevated liver enzymes and moderately increased odds for liver fibrosis/cirrhosis. SZ individuals also displayed higher liver enzymes, more frequent liver fibrosis/cirrhosis and primary liver cancer. Disease modifiers associated with significant liver fibrosis included male sex, age (≥ 50 years), BMI of ≥ 30, and the presence of diabetes [3].

Schneider et al. analyzed data from the European Alpha-1 Liver Cohort to compare features of adults with and without the MZ genotype among persons without pre-existing liver disease [5]. [5More than 400 MZ subjects underwent a comprehensive evaluation of liver phenotype using liver stiffness measurements and liver biopsies to define histologic and biochemical features of this genotype. In addition, levels of serum transaminases were measured. Relatively minor changes were found between noncarriers and MZ subjects. Adults with the MZ genotype were found to have lower levels of serum transaminases, fewer AAT inclusions in liver, and lower liver stiffness than those with the ZZ genotype, but still higher than adults without the Z variant. Biopsies showed that AAT accumulation is variable among MZ individuals but that accumulation increases with age in MZ population. The more fibrosis, the more inclusions, suggesting a vicious cycle between injury and AAT accumulation [5]. With regard to modifiers promoting liver disease, metabolic risk factors such as obesity or diabetes as well as excessive alcohol consumption are the best-established triggers [6]. Consistent with this observation, the MZ genotype promotes the development of liver fibrosis/cirrhosis in several liver diseases [4, 7]. Further, because MZ subjects with cirrhosis decompensate faster than noncarriers, early evaluation is important to improve transplant-free survival [8].

In sum, most MZ individuals never develop clinically significant liver disease. However, they are sensitive to additional hits such as obesity, diabetes, and alcohol consumption. A primary goal therefore should be to identify and advise MZ individuals about their relative risks early on.

Discussion focused on the following issues. A high BMI is a risk factor that applies to all patients, not just MZ individuals, and is a complex problem because of multiple contributing factors (e.g., diet, sedentary lifestyle). Inclusions and globules can be found in the explants of MZ patients and pathologists will likely not be able to differentiate between MZ and ZZ individuals based on their histologic presentation. Inflammation and bacterial infection occurs frequently in patients with decompensated liver cirrhosis and may drive the development of liver failure in MZ patients. Final discussion centered on how to identify those at risk, whether ZZ or MZ. The presence of disease modifiers and cofactors in these populations, such as obesity and elevated serum transaminase levels, might be indicators of risk or underlying liver disease. It was suggested that known MZ status should be considered a primary indicator of elevated risk for end-stage liver disease. Studies of larger cohorts are needed to develop more accurate predictions.

Heterozygosity of the Alpha 1-Antitrypsin Pi*Z Allele and Risk of Liver Disease

Aaron Hakim, MD, MS, Beth Israel Deaconess Medical Center, Boston

A 32-year-old male patient with decompensated cirrhosis who was listed for transplant was described. He lacked traditional risk factors for liver disease but presented with ascites and esophageal varices. Conventional hepatology evaluation was unremarkable; quantitative AAT testing was within normal limits. The patient was enrolled in a whole-exome sequencing study and was found to have a heterozygous mutation in *SERPINA1* at the Z allele. This case highlights the need to identify heterozygous Z allele individuals and address risks. Genomic screening offers one promising approach at the population level.

The definition of the cirrhosis phenotype by ICD-10 codes in the UK Biobank showed that patients with cirrhosis were older, had elevated BMI, polypharmacy, and expected changes in liver synthetic function markers and biochemistries. Much of the genetic basis of chronic liver disease has not been described. To identify the genetic loci that regulate the risk of liver injury, Hakim and colleagues performed genome-wide association studies on circulating levels of alanine aminotransferase (ALT), aspartate aminotransferase (AST), alkaline phosphatase (ALP) and total bilirubin among a large sample of participants in the UK Biobank [9]. The Z allele was associated with elevated markers of liver injury and cirrhosis by ICD-10 codes.

Approximately 5% of subjects with European ancestry are Z heterozygotes. The magnitude of the effect of the Z allele on these liver phenotypes was similar to other known liver disease variants in PNPLA3 and TM6SF2. These findings confirm that the Z allele confers increased risk of liver injury. The same is true in homozygotes but with larger effect sizes. The ALT measure is a useful endophenotype because it is a quantitative measure of liver injury where variations even within the reference range are predictive of disease. Gene-environment interactions such as BMI also amplify the effect of the Z allele on ALT. Hakim and colleagues replicated these findings in a hospital-based cohort of unrelated European ancestry subjects with and without cirrhosis in the Massachusetts General Brigham Biobank.

In conclusion, Z allele heterozygotes may have increased risk of liver injury and cirrhosis. These findings suggest that the *SERPINA1* Z allele could be a potential therapeutic target and, if so, could provide benefit to both the ZZ population and a select subset of heterozygous patients. If the latter subset can be accurately identified, mitigation strategies could be used to silence protein AAT expression in the liver, decrease the burden of globules, and potentially minimize fibrosis.

Participants discussed whether the lower cost of whole exome sequencing might encourage its use as a screening tool for the Z allele. To date, most cohorts primarily include MZ patients who are symptomatic, so they are not a representative sample. Although new population data are informative, they have limitations. Studies to date have focused on relatively homogenous European ancestry populations, requiring future studies of individuals with more diverse genetic ancestries. Further, gene/gene interactions mediating liver disease remain to be identified for Z allele heterozygotes. Finally, the role of gene-environment interactions in mediating liver damage in Z allele heterozygotes requires further investigation, such as the interaction of the Z allele with COPD, alcohol use, and inflammation. The Z allele might only be a piece of the puzzle that increases levels of AAT. All of these second “hits” are likely to be additive or result in a synergistic outcome. Increased knowledge of the relative roles of these contributing factors in terms of risk and length of exposure would be useful in genetic testing and counseling of families with a history of the Z allele. The pathways to cell death are likely to be the same despite the cause, which could yield alternative therapeutic strategies.

## Session II: Relative risk of lung disease among heterozygotes: What can cohort studies tell us about the heterozygous disease state?

*Relative Risk of Lung Disease Among Z Heterozygotes*

Tomás Carroll, PhD, Royal College of Surgeons in Ireland

Ireland has collected longitudinal cohort data on AATD for decades, where prevalence of the Z allele is relatively high: 1/25 for MZ, 1/424 for SZ, and 1/2,104 for ZZ. Globally, it is estimated that less than 10% of those with the ZZ genotype have been diagnosed. However, on the island of Ireland, 16% of the estimated 3,000 ZZ individuals have been identified due to targeted testing efforts. The Irish National Alpha-1 Targeted Detection Programme has tested more than 24,000 people since 2004 of whom 6,000 had severe or moderate AATD [10]. Critically, many cases were identified within large families, offering an opportunity to determine whether Z heterozygotes are at greater risk of COPD. A 2004 meta-analysis found that while case-control studies had shown increased rates of COPD in MZ individuals, this finding was not confirmed in cross-sectional studies [11]. The studies reviewed had several limitations including: selection bias; small sample sizes; inconsistent methods for diagnosing COPD; incomplete smoking histories; lack of rigorous control for age, sex, and ethnicity; and lack of clarity in the COPD subtype. In addition, many analyzed hospital cohorts. As such, the data did not capture how many MZs are in the community and whether they are symptomatic.

The Irish MZ Family Study was a 2-year Irish/U.S. collaboration and sought to reduce ascertainment or referral bias by excluding the MZ proband in each family from final data analysis. Probands can represent “outliers” as they are identified first and often because of exceptionally severe disease that is not representative of all MZ individuals. The final cohort included 239 individuals and crucially compared 99 MM individuals to 89 MZ siblings. Spirometry data showed greater evidence of COPD in the MZ individuals compared to their MM siblings. These findings confirmed that MZ heterozygotes have significantly more airflow obstruction and COPD than MM individuals [12]. In addition, analysis of the data found that COPD risk was driven by cigarette smoke exposure. MZ never smokers had similar post bronchodilator FEV1% predicted when compared to MM never smokers. However, MZ ever smokers had reduced lung function compared to MM ever smokers, and this reduction was more pronounced in those with heavy smoke exposure (> 20 pack year). A working hypothesis to explain these findings is that never smoker MZ individuals have sufficient AAT levels to protect their lungs, but in MZ smokers the already reduced AAT level is further diminished by cigarette smoke-induced oxidation of AAT, leading to a greater imbalance between AAT and neutrophil elastase [13].

Carroll and colleagues then performed a second prospective family-based study of lung risk, this time in the SZ genotype. Again, the study was designed to reduce referral bias by excluding SZ probands in each family. Unlike the prior MZ study, GOLD Stage 2–4 was not part of the inclusion criteria for probands. Forty-four Irish families and 166 subjects participated from 2016 to 2019, of whom a total of 82 were SZ and 46 normal risk individuals who were either MM or MS.

The data showed that SZ never-smokers demonstrated no increased risk of COPD when compared to never smoking controls. However, smoking combines with the SZ genotype to significantly increase the risk of COPD compared to ever-smoking controls. A history of smoking alone was not associated with greater lung function decline in SZs, suggesting that stopping smoking prevents lung disease from getting worse. In sum, there was strong evidence for the SZ genotype as a risk factor for COPD, but only among smokers. A second follow-up registry study found that the SZ genotype resembles MZ in terms of lung risk; that is, SZ should be regarded as a moderate deficiency phenotype, akin to MZ. These findings emphasize the need for early diagnosis and behavior modification as the most effective interventions against the development of lung disease in a nation where there are approximately 40,000 MZ smokers with an increased risk of lung disease [14].

Discussion focused on the possibility that MZ individuals who develop COPD might have other genetic factors that predispose them to risk. As such, whole exome sequencing could elucidate the possible role of other genes. This has been the case in non-AATD COPD, for which there are many genetic variants and polygenic risk scores. It was suggested that educating those at risk about the potential harm caused by exposure to second-hand smoke and occupational irritants is also important. Smoking cessation programs for people at risk due to both severe and moderate AATD in Ireland have had a clear positive effect, with a strong impact on quit rates [15].

*SERPINA1 MZ Associations in UK Biobank*

Ian Hall, FMedSci, University of Nottingham

The UK Biobank includes data on more than 500,000 individuals aged 40–69 at time of recruitment. A large, population-based study was conducted to determine Z allele effects on more than 2,400 phenotypes in the bank [16]. Sex, height, smoking status, and genotyping data were obtained. Of those directly genotyped for the Z allele, 3.94% were found to be heterozygous and 0.0314% were homozygous, much lower than expected under Hardy-Weinberg equilibrium in all samples. The full UK Biobank dataset contains 141 homozygotes, of which 20 had an ICD code diagnosis. These findings suggest underdiagnosis in this population.

Further analysis showed that 58 of 93 individuals homozygous for the Z allele did not have spirometrically defined COPD. Further, four out of 15 homozygous ever-smokers over the age of 60 did not have spirometrically defined COPD. These findings suggest that individuals with the Z genotype but without COPD may have a more protective genetic profile across other genomic regions.

Those heterozygous for the Z allele exhibited higher forced expiratory volume (FEV1) but no association with FEV1/forced vital capacity (FVC) or FVC compared to wild type. In a stratified analyses of UK Biobank never- and ever-smokers, Hall and colleagues found that heterozygosity for the Z allele was associated with a large increase in FEV1 and increased FEV1/FVC in never-smokers, but not in ever-smokers, with a small increase in COPD risk in ever-smokers. Further, Z allele heterozygosity was strongly associated with increased height. Heterozygosity was also associated with higher height-adjusted FEV1 and FEV1/FVC in nonsmokers. In other words, increased lung function in the MZ genotype is driven by height (the height effect obscures effects on lung function). This protective effect was not found in ZZ genotypes. These findings also show for the first time that sex modifies the association of the Z allele on lung function.

A phenome-wide association study (PheWAS) was then conducted to ascertain associations between the Z variant across a large number of different phenotypes. Z allele homozygosity was associated with increased hemoglobin, hematocrit, and red blood cells, as well as optic neuritis and pancreatitis. Strong associations were found between Z allele heterozygosity and several clinical features. Heterozygosity was also associated with several biomarkers, including higher ALT, albumin, ALP, AST, bilirubin, calcium, sex hormone-binding globulin, and testosterone.

These data provide some mechanistic insights. First, COPD risk among heterozygotes belies a complex relationship between height, lung function, and smoking. Non-smoking heterozygotes have increased height and higher lung function. Smoking heterozygotes have an increased risk of COPD. The mechanism underlying the height effect is unclear. However PheWAS studies did show an association with bone metabolism, osteoarthritis, osteoporosis, and also with testosterone, possibly indicating an effect on skeletal maturation. The data also suggest important effects on biliary tract disease in heterozygotes, small but significant effects on risk of heart disease and blood pressure, and extensive effects on a range of circulating biomarkers. New Olink data could help unravel underlying mechanisms that will extend our understanding of *SERPINA1*-regulated pathways.

In sum, the UK biobank continues to provide a useful resource to explore general population effects of the alleles. The expected effects were observed for homozygous individuals. Of interest, heterozygote smokers have an increased risk of COPD but non-smokers have better lung function, probably due to increased height. Further, heterozygotes have increased risk of a number of other conditions, including gallbladder and bone disease. The proteomic associations with the Z allele are extensive and probably indirectly drive some of the pathology.

Discussion focused on the plausible explanations of the height effect, including genetic ancestry. The data also suggest a role of *SERPINA1* in the vasculature based on differences found with regard to ischemic heart disease and migraine.

*Alpha-1 Antitrypsin MZ Heterozygosity is an Endotype of COPD*

Auyon Ghosh, MD, MPH, SUNY Upstate Medical University

Seminal work by Hersh et al. demonstrated the increased risk of COPD among individuals heterozygous for AATD, confirming the observation that MZ individuals are often identified with established COPD [11]. However, an MZ diagnosis currently has no effect on management.

Ghosh and colleagues identified differences in clinical characteristics between MZ and MM COPD by examining longitudinal and survival differences [17]. They further explored the differences in gene expression in lung tissue and whole blood. Subjects were obtained from three cohorts: COPDGene (smokers and non-smokers with and without COPD), ECLIPSE (COPD and control), and the Lung Tissue Research Consortium (LTRC, ECLIPSE, and COPDGene subjects were followed for a variable number of years—5, 8, or 10. *SERPINA1* genotype was determined from whole genome sequencing.

Compared to MM individuals, MZ heterozygous individuals had reduced median post-bronchodilator FEV_1_ (% predicted), FEV1/FVC ratio, and FEF25-75 (% predicted). This effect was nullified in never-smoking and accentuated in ever-smoking MZ individuals. In differential expression analyses, no differentially expressed genes were found in whole blood in the COPDGene cohort and only one differentially expressed gene, *PGF*, was found in lung tissue in the LTRC cohort. Deconvolution of immune cell types revealed a higher neutrophil count in MZ COPD lung tissue. Although MZ individuals with COPD have worse lung function and increased emphysema on chest CT scans, no difference in mortality was observed; a finding discussants suggested might be due to some cardiovascular protective effect of the Z variant. A next step is to search for genetic modifiers that might explain variability among these individuals.

Longitudinal Analysis of Clinical Outcomes in Pi*MZ Alpha-1 Antitrypsin Deficiency Smokers from the SPIROMICS Cohort

Igor Barjaktarevic, MD, PhD, David Geffen School of Medicine at UCLA

Since the discovery of severe AATD as a genetic risk factor for lung disease, there has been controversy about whether MZ individuals are also at some increased risk for lung disease. Al Ashry and Strange reviewed studies that compared the decline of lung function over time among MM and MZ individuals [18]. They concluded “Carefully designed family studies show an increased risk of emphysema in MZ smokers. This is supported by the rapid decline in lung function of MZ individuals when compared to the general population after massive environmental exposures.” The analysis also found that risk of emphysema is more pronounced in case-control than population-based studies, perhaps due to inadequate power. Across studies the smoking data are not always clear.

The MZ genotype has been studied extensively in the COPDGene and ECLIPSE cohorts, as described by Ghosh above. Cross-sectional data from Foreman et al. suggested that MZ heterozygous individuals who smoke are at increased risk for COPD, have lower FEV1, and have more emphysema and air trapping [19]. After adjusting for the principal components of genetic ancestry, Ghosh et al. found no difference in the change in the FEV1 (% predicted) or adjusted lung density from baseline [17].

The Subpopulations and Intermediate Outcome Measures in COPD Study (SPIROMICS) is an observational cohort of individuals ages 40 to 80 years that provides an opportunity to analyze MZ individuals. Close to 3,000 individuals with COPD or at risk of COPD, along with healthy controls, have been followed over the past 10 years in 12 large U.S. academic centers. The goal is to recognize the biomarkers that differentiate individuals with COPD and enable adjusting management and predicting outcomes in this population. Participants were enrolled as healthy never-smoking controls, or those with a smoking history of more than 20 pack-years who are either at risk of COPD or are diagnosed with COPD.

In SPIROMICS, DNA samples were sequenced in a 16.9 kB region of *SERPINA1* [20]. Among the 1,947 individuals with a history of smoking > 20 pack-years who consented to genetic testing, 1,856 (95.2%) were MM, 79 (4.1%) were MZ, 9 were ZZ, 3 were SZ, and 2 were SS. Of interest, all 79 of the MZ individuals were current or former smokers. Based on these data, Barjaktarevic and colleagues hypothesized that when compared to MM ever-smokers, MZ ever-smokers will have more progressive lung disease as evidenced by higher annual rate of decline in FEV1, more significant loss of lung density, and more prospective exacerbations [20]. They conducted a cross-sectional evaluation of baseline lung function and disease state in all participants and evaluated longitudinal outcomes that included annualized rate of change in FEV1 and change in functional performance status. Participants were followed longitudinally over approximately 5 years.

More COPD was found among MZ then MM individuals. MZ individuals also had more emphysema and lower mean lung density on chest CT scans. However there was no difference in airway wall thickness, small airway disease, or air trapping. Interestingly, the annual rate of FEV1 decline was similar for both groups (29 vs. 30 ml) and there was no significant difference in the annualized rate of change in other clinical outcomes, including mortality, although PiMZ individuals exhibited a trend toward more emphysema progression and higher mortality. Among actively smoking PiMZ participants, there was a trend toward more emphysema progression over the study follow up period (*P* = 0.07) but there was no statistically significant difference compared to PiMM smokers. Rapid decliners were numerically more often active smokers with more emphysema and a higher rate of baseline exacerbations. Taken together, the data confirmed previous findings that PiMZ smokers have worse lung function and more emphysema when compared to PiMM smokers, nevertheless, despite consistent trends toward more emphysema progression, Barjaktarevic and colleagues could not show statistically significant differences in longitudinal outcomes.

Discussion focused on the importance of enrolling younger people into trials to better assess risk progression over time. Relatively small numbers of participants limits the ability to draw more generalizable conclusions from this analysis. Future research should focus on MZs who are never-smokers to establish baseline risk and to better define and identify emphysema in this population because of its heterogeneity. More insight might be gained by combining study cohorts and following them longitudinally. However, measurements of lung function differ across AATD and COPD studies and endpoints differ. Finally, the MZ COPD population is a diverse group so it is important to identify an appropriate control group. More homogenous cohorts could better isolate an effect.

## Disease mechanisms and Z heterozygosity

*Disease Mechanisms and Z Heterozygosity: Evidence from Human Macrophages*

Gerry McElvaney, MD, Royal College of Surgeons in Ireland

AAT acts primarily to inhibit neutrophil elastase (NE) in the lung, thus protecting lung tissue from proteolytic degradation. AAT also inhibits other neutrophil-derived proteases, such as cathepsin G (Cath G) and proteinase-3. There is a significantly higher burden of neutrophils in the lungs of individuals with AATD compared to healthy individuals.

The role of NE in emphysema has been described for some time. Shapiro et al. demonstrated that when NE-deficient (NE-/-) mice and wild-type (NE+/+) littermate controls were exposed to long-term cigarette smoke, mice that were deficient in NE were significantly protected from the development of emphysema [21]. Previously, Hautamaki et al. found that in contrast to wild-type mice, macrophage elastase-deficient (MME-/-) mice did not have increased numbers of macrophages in their lungs and did not develop emphysema when exposed to cigarette smoke [22]. Hautamaki et al. concluded that macrophage elastase is likely sufficient for the development of emphysema in chronic smokers. In 2003, Churg et al. reported that both macrophage elastase (MMP-12) and NE play a role in acute cigarette smoke-induced connective tissue breakdown, the precursor of emphysema [23].

Geraghty et al. postulated that NE could induce expression of cathepsins and MMPs in human monocytes [24]. They found that NE exposure resulted in monocytes producing significantly greater amounts of cathepsin B and MMP-2, and that in an NE -/- mouse model, infection with Pseudomonas aeruginosa resulted in less lung destruction and decreased MMP-2 and cysteinyl cathepsin expression than that seen in NE wild type (+/+) mice. These results suggest there is a hierarchy among proteases in which a serine protease, such as NE upregulates expression of MMPs and cysteinyl cathepsins. To better understand this, Geraghty et al. assessed levels of cysteinyl cathepsin and MMP activity in bronchoalveolar lavage (BAL) fluid from AATD patients, pneumonia patients, and healthy controls [24]. BAL fluid from patients with pneumonia and those with AATD containing free NE had increased cathepsin B and MMP-2 activity compared with BAL fluid from healthy volunteers. The addition of AAT to BAL fluid from patients with pneumonia greatly reduced NE-induced cathepsin B and MMP-2 expression in macrophages in vitro. Augmentation therapy administered via aerosol to AATD patients also reduced cathepsin B and MMP-2 activity in BAL fluid in vivo.

In addition to being a plasma protease inhibitor, AAT is also a multifunctional protein that modulates immunity, inflammation, proteostasis, apoptosis, and cellular senescence. Protein phosphatase 2A (PP2A), a serine-threonine phosphatase, regulates similar biologic processes and has been shown to play a role in chronic obstructive pulmonary disease. Geraghty et al. confirmed that, given their common effects, AAT modulates PP2A to alter macrophage-produced cytokines [25].

AAT is also synthesized by monocytes. Carroll et al. found that the unfolded protein response of *SERPINA1* is activated in monocytes from ZZ individuals [26]. This contributes to an inflammatory phenotype with ZZ monocytes exhibiting enhanced cytokine production when compared with MM monocytes. The amplified inflammatory response generated by accumulated aberrantly folded AAT in circulating blood cells could explain the causes of lung inflammation in those with a Z allele. Future research is needed to elucidate what activity is occurring in MZ macrophages. In general, the question is whether MZs have an increased risk for lung disease due to insufficient less effective AAT in the extracellular spaces of the lung, or does the intracellular retention of Z AAT in lung cells rendering them pro-inflammatory also contribute? To elucidate this would require following a cohort for several years with regular blood tests, pulmonary function tests, CT scans, and analyses of BAL fluid to evaluate proteases and cytokines and levels of intracellular stress.

Discussion items included questions about the role of free polymers in the lungs, whether studies of explanted lungs would be informative, the relative role of AAT versus environmental effects on mitochondrial misfunction in MZ, and whether what is being observed is an inflammatory or proinflammatory effect.

*Modeling ZAAT-Driven Gain-of-Function Toxicity in MZ Heterozygous Patient iPSC-Derivatives*

Joseph Kaserman, MD, Boston University

Despite growing clinical evidence supporting increased MZ liver disease risk, the lack of current model systems that faithfully reproduce human MZ hepatocyte biology has been a significant limitation in performing mechanistic studies or directly comparing it to the more highly characterized ZZ pathobiology. Transgenic “PiZ” mice co-express murine AAT with multiple copies of the human Z allele, while immortalized cell lines engineered to overexpress Z or MAAT fail to capture patient-to-patient genetic heterogeneity or hepatocyte-specific biology that could contribute to risk among individuals [27–31]. Given the high prevalence of Z heterozygosity in the US population, there is a critical need to establish model systems that accurately reproduce human MZ hepatocyte biology and determine how heterozygosity can induce injury.

Work generated by multiple groups has shown that ZZ patient-specific induced pluripotent stem cell (iPSC)-derived hepatocytes (iHeps) capture cellular features of ZZ-associated liver disease pathogenesis [32–39]. To test the hypothesis that a single Z allele is sufficient to induce human liver injury, Kaserman et al. engineered syngeneic MZ and MM iPSCs from highly phenotyped ZZ patient derived iPSCs using CRISPR-Cas9 in combination with single-stranded oligodeoxynucleotide donor templates. Three genetically distinct ZZ iPSC lines (PiZZ1, PiZZ6, and PiZZ100) were targeted, generating three syngeneic sets of ZZ, MZ, and MM iPSCs for a total of nine iPSC lines [34]. Following specification to the hepatic lineage Kaserman et al. found that MZ iHeps had evidence of altered AAT secretion kinetics resulting in an associated downstream metabolic dysregulation, impaired mitochondrial function, and cellular heterogeneity. Transcriptionally, both MZ and ZZ iHeps had disruption of their global transcriptome including perturbations in key transcription factors and enzymes associated with lipid metabolism and the urea cycle. They then extended their findings characterizing the metabolome using liquid chromatography-mass spectrometry combined with extraction methods to enrich for both lipid and amide metabolites in ZZ, MZ, and MM iHeps. MZ and ZZ iHeps had 132 and 128 differential metabolites respectively compared to MM iHeps, including reduced levels of ATP, C2 acylcarnitine, long chain acylcarnitines, and urea cycle metabolites. These findings highlighted Z AAT-associated metabolic dysregulation in fatty acid oxidation and the urea cycle. Given the alterations in fatty acid metabolism in MZ iHeps they then tested whether this was associated with mitochondrial derangements. They found that MZ and ZZ iHeps both demonstrated significant morphological disruption relative to a genetically controlled MM iHep comparator and that this was accompanied by reduced mitochondrial function when analyzed using respiromics [34].

Finally, single-cell RNA sequencing was used to collect expression data from syngeneic ZZ, MZ, and MM iHeps generated from the PiZZ1 background together with a second ZZ sample from the PiZZ6 background. Bioinformatic analysis demonstrated that MM iHeps clustered independently while MZ and ZZ iHeps clustered together. The bulk of the Z expressing iHeps were contained in two distinct clusters characterized by expression profiles that were either (1) “pro-fibrotic” and less metabolically active, or (2) “secretory” and more synthetically active. They then applied gene module scores specific for each branch of the unfolded protein response (UPR) finding that iHeps within the pro-fibrotic cluster demonstrated upregulated PERK and downregulated ATF6 and IRE1 branch expression while iHeps within the secretory cluster exhibited the inverse expression pattern [34].

In sum, these data demonstrate that syngeneic MZ iPSCs engineered from highly phenotyped ZZ individuals with AATD can effectively model MZ hepatocyte heterozygosity and that a single Z allele can disrupt homeostatic hepatocyte functions. MZ iHeps have an intermediate phenotype but share with the ZZ cells alterations in AAT processing, metabolic and mitochondrial dysregulation, and cellular heterogeneity with UPR branch-specific activation. In discussion, Dr. Kaserman highlighted that the MZ iHeps used in this study were generated from ZZ individuals with severe clinical liver disease and thus could have genetic cofactors that would result in more significant cellular dysfunction. He discussed future experiments comparing MZ iHeps derived from ZZ individuals protected from liver disease that could be informative in this regard. Dr. Kaserman also discussed that comparisons to primary hepatocytes from MZ individuals would be informative but are logistically challenging to obtain, illustrating a potential rationale for utilizing iPSC-derived hepatocytes.

*Polymers, Heteropolymers, and the Heterozygous State*

David Lomas, MD, University College London

The Z variant of AAT promotes proteasomal degradation and the formation of polymers. Despite their retention in the endoplasmic reticulum (ER), AAT polymers do not initiate the unfolded protein response. Instead, they are sequestered into ER-derived inclusion bodies within the hepatocytes that are associated with liver disease. The lack of circulating AAT promotes dysregulation of NE and subsequent tissue destruction and emphysema. As such, AATD can be described as a disease of polymerization. Plasma deficiency of AAT is in fact proportional to the rate of polymerization in all but the null mutants [40, 41].

To determine which model best describes the pathological (mutant) polymers that can form in AATD, Lomas et al. completed a structural characterization of polymers from explant liver tissue of ZZ individuals who had undergone transplantation [42]. Polymers were isolated from the explanted liver tissue of ZZ homozygotes and subjected to single-particle analysis of negative-stain electron micrographs. The results revealed structural equivalence between heat-induced and ex vivo polymers. Inter-subunit linkage occurs via a carboxyl-terminal domain swap between molecules of AAT [42].

By co-expressing two AAT variants, each modified by a different tag, Lomas and colleagues developed cell models that replicate heterozygote AAT deficiency. They showed that Z AAT forms heteropolymers with S and with rare mutants, but to a lesser extent with the M variant. Increased intracellular accumulation of AAT variants was observed when co-expressed with Z AAT, suggesting a dominant effect of the Z allele. That is, the presence of Z AAT increases the intracellular retention of both the S and M form of AAT [43, 44].

These results provide evidence of intracellular co-polymerization of AAT mutants and contribute to understanding the risk of liver disease in SZ and MZ heterozygotes. Z and MZ polymers are likely a co-factor for liver disease with alcohol consumption and fat providing a second hit. Thus, MZ heterozygotes may benefit from the same polymer blocking therapy as ZZ homozygotes, such as siRNA or small molecules.

Dr. Lomas and colleagues have addressed several challenges in their efforts to develop a small molecule polymer blocker that stabilizes Z AAT. For example, the drug target is a highly mobile folding intermediate in the ER, large protein-protein interactions have to be prevented, and oral dosing greatly restricts the chemical space that can be explored. Moreover any absorbed drug needs to saturate the low level of circulating Z AAT before it can access the liver. Using a DNA-encoded chemical library, they conducted a high-throughput screen to identify small molecules that bind to and stabilize Z AAT. The resulting lead compound blocks Z AAT polymerization in vitro and increases the secretion of Z AAT threefold in an iPSC model of disease [45]. It also increases the secretion of Z AAT in a mouse model of AAT deficiency. Blocking polymerization provides a novel strategy to prevent disease and may be particularly useful in MZ heterozygotes as the small molecule binds more readily to Z than M AAT. Questions remain about appropriate dosing and duration of treatment.

Discussion focused on the challenge of extrapolating dosing from mouse to human for optimal potency. Questions were raised about potential toxicity if active Z is released into the circulation. Dr. Lomas noted that toxicity has not been observed, as the released Z is likely to be inactive.

## Session IV: Treatment and the heterozygous state

*How Should We Treat MZs with COPD?*

Charlie Strange, MD Medical University of South Carolina

Despite COPD’s high prevalence, little research has been conducted on therapy for specific endotypes. The trend in emerging therapeutic devices and biologics for COPD is to identify treatment approaches that are matched to the pathobiology of disease. In this context, no studies have ever been done targeting the lung disease of MZ individuals.

COPD’s common endotypes include emphysema, chronic bronchitis, bronchiectasis, and incompletely reversible asthma. These endotypes can each have frequent exacerbation populations and some have existing therapies that are partially effective. The measurement tools used to assess outcomes are crude and the timelines of progression are long. There is little correlation between CT lung density used to assess emphysema and FEV1 used to assess airways obstruction. Further, many patients with these endotypes can have normal spirometry. Although there are guidelines for workup and treatment of these endotypes, none are specific to COPD occurring in MZ patients.

Therefore, to extend specific therapeutics from the ZZ population to a more mildly affected MZ population without any clinical trials is not evidenced-based medicine. Instead, pulmonary specialists have focused general COPD care on the MZ population with COPD to include assessment of eosinophilic airways disease and better determine the frequency of exacerbations. This is critical as research has demonstrated that exacerbations are common in AATD patients with lung disease [46]. Exacerbations of COPD are the most common reason for hospitalization and are associated with decreased health-related quality of life and increased risk of mortality. Management of exacerbations in the acute stage aims to relieve dyspnea, reduce airway inflammation, improve lung function, and eradicate infections. Maintenance focuses on reducing the risk of a new exacerbation.

One problem is that both patients and healthcare providers fail to accurately determine the symptoms of a COPD exacerbation. A recent survey of AlphaNet subscribers provided self-reported data on treatment approaches used by AATD patients: nonpharmacological (e.g., smoking cessation, exercise), pharmacological (e.g., pharmacotherapy for smoking cessation, inhaled and other treatments) and assessment and education (e.g., inhaler techniques, adherence to therapy) [47]. The major finding of the survey was that patients underreport symptoms and healthcare providers fail to record the majority of clinical worsening events. In response, an Exacerbation Management Toolkit was developed that may help patients recognize their triggers, optimize their medications, and make an exacerbation plan to share with their healthcare provider. This missed opportunity to treat exacerbations more aggressively in MZ patients can be rectified.

The potential value of high-dose augmentation therapy in those with ZZ AATD has been the focus of the Study of ProlAstin-c Randomized Therapy with Alpha-1 augmentation (SPARTA). The premise is that clinical outcomes of AATD can be improved by weekly administration of alpha1 protease inhibitor. Initiated in 2013, 341 subjects have been enrolled to date. The study design is a randomized, placebo-controlled trial assessing the efficacy and safety of two separate doses of Prolastin-C (60 and 120 mg/kg) administered weekly over 3 years in AATD patients aged 18 to 70 years with clinical evidence of pulmonary emphysema [48]. If there proves to be a difference in emphysema progression between ZZ patients augmented with 60 mg/kg and 120 mg /kg, then the blood levels achieved in the high-dose group would provide biochemical plausibility to support AAT augmentation in MZ individuals. Currently, however, several conditions would have to be met to treat a non-smoking MZ individual with augmentation. They would have to be genotyped, have emphysema-predominant COPD, show disease progression despite optimal non-augmentation therapy, and there would have to be some evidence of efficacy as demonstrated in clinical trials.

Subsequent discussion addressed the challenges ahead. For example, augmentation therapy is not licensed in the United Kingdom. Not only does this have clinical implications but it also restricts the feasibility of attaining the statistical power needed in this rare disease to document incremental improvement. An additional challenge arises from disease heterogeneity and its implications for meeting regulatory requirements, which typically require homogeneous cohorts in order to isolate and identify effect. Further the definitions of phenotypes and endotypes differ across studies and clinical settings. Because AATD is a rare disease, it is essential to include as many individuals as possible in studies, which necessitates careful and consistent endotyping. Finally, improved understanding of the nature and frequency of exacerbations in both MZ and ZZ populations will inform the design of trials and treatment approaches.

Augmentation Therapy in Pi*MZ: What Does the Clinical Trial Look Like?

Bradley Drummond, MD, University of North Carolina at Chapel Hill.

The key components of clinical trials are the target population, intervention, primary outcome, anticipated effect size, and trial duration. The RAPID study is illustrative of the type of trial that includes these essential components [49]. RAPID was a multicenter, double-blind, randomized, parallel-group, placebo-controlled trial of administration of weekly alpha1 protease inhibitor in patients with AATD. Participants were non-smokers aged 18–65 years with severe AAT deficiency and FEV1 35–70% predicted. The intervention randomly assigned patients to receive the treatment intravenously 60 mg/kg per week or placebo for 24 months. The primary outcome was annual rate of decrease in 15th percentile lung density (PD15) as summed total lung capacity (TLC) and functional residual capacity (FRC) combined and separately. The sample size was 180, which was estimated to provide sufficient statistical power to establish significance based on anticipated treatment effect size.

Chapman et al. concluded that measurement of lung density with CT at TLC alone provides evidence that augmentation slows progression of emphysema. Of note, this finding could not be substantiated by lung density measurement at FRC alone or by the two measurements combined due to greater variance in FRC measurements. These findings support the value of augmentation treatment to preserve lung parenchyma in patients with emphysema secondary to severe AATD and ZZ genotype. Importantly, the trial demonstrated that PD15 is the optimal outcome for assessing lung density as an indicator of pathophysiology, informing a key component of clinical trial design for MZ studies.

Other potential clinical trial outcome measures were discussed and felt to be problematic as primary outcome measures. The change in FEV1 between MZ and MM individuals is small; longer-term follow up with larger samples sizes is needed to demonstrate impact of augmentation therapy among MZ individuals. Incident COPD and COPD-related outcomes are also problematic as primary outcomes. As discussed above and published in Molloy et al., differences in study design can introduce or ameliorate selection bias that influences key study findings [12]. This and other studies have underlined the limited value of health-related quality of life and acute exacerbations of COPD as primary outcome measures. In contrast, CT metrics (i.e., TLC PD15) are more standardizable, have lower measurement variation, permit comparison to ZZ clinical trial data, identify early disease, and are a sensitive measure of treatment effects.

Further research is needed to better understand the significance of PD15 in MZ heterozygotes, especially among never-smokers, annual changes in MZ lung density over time, and the effect of augmentation on PD15 in MZ heterozygotes. The RAPID trial results highlighted several considerations for inclusion criteria that remain unclear in the MZ population: former versus never smokers; preserved versus impaired lung function; evidence of CT emphysema; and selection based on AAT level. Ideally, a trial should last at least 2 years to maximize detection of impact of augmentation therapy. There is an exponential increase in sample size as treatment effects decrease, such that large sample sizes (300–800 participants) may be required. Dosing, duration, and sample size is not estimable based on current data. Thus, a pragmatic strategy to inform definitive clinical trial design may be prudent, consisting of a 1- to 1.5-year pilot study with the inclusion criteria cited above and dosing of 120 mg/kg weekly to ensure maximal effect of augmentation therapy is observed. Outcomes of interest include PD15 at TLC, pharmacokinetics, FEV1, and patient-reported outcomes.

During discussion participants raised other design issues. Discussions included the appropriate age for inclusion and exclusion. Given concern for enrolling after disease has already started, eligibility should include individuals who have attained maximal lung function (i.e., 40 years of age). Another challenge discussed was how best to select and stratify enrollment. Factors considered included baseline biomarkers of inflammation, smoking history, and alpha-1 antitrypsin level. There is value in understanding treatment effects on MM homozygotes as well, especially those with COPD or some evidence of end-organ disease as control groups, as they would likely have similar changes in lung function to non-smoking MZ individuals. Other considerations are the need to conduct dose escalation studies and improve measurement of inflammation in the lung.

*Panel Discussion*

Virginia Clark, MD; Bradley Drummond, MD; Monica Goldklang, MD; Peg Iverson, Charlie Strange, MD; Jeffrey Teckman, MD

Panelists discussed the current state of and prospects for treatment strategies for MZ heterozygotes. Several therapeutics are in development to treat the liver disease associated with AATD by removing the Z protein from the liver. As many as 15 drugs are in the pipeline, all aiming to enroll patients with the ZZ genotype, so there is competition for subjects. For this reason it is critical to also study liver disease in the MZ genotype because the signals are there and the comorbid states are less common. The challenge is in finding the numbers needed: of the 3.3 million people with the MZ genotype, 98% are asymptomatic, so they are essentially undetected.

Treatments for lung disease in the MZ population are also being explored. Several experimental NE inhibitors have been tested in preclinical and clinical studies of different conditions of inflammatory lung injury such as pneumonia, with contrasting results. Elastase inhibitors are likely to be more practical and less expensive therapeutic approaches for this population, which has less severe lung disease, if any. The lack of good data on the value of augmentation therapy for MZ lung patients precludes its use until proven safe and effective in this population. If MZ heterozygotes are found to have inflammatory problems similar to those with the ZZ genotype, then they might benefit from augmentation therapy.

In some ways, the genotype is the low-hanging fruit, but treating the genotype is not likely to be the most effective or beneficial approach. Endotypes and phenotypes are more likely to guide treatment strategies given the heterogeneity of the MZ genotype. Studying MZs within a given disease category (e.g., COPD, asthma) might be more informative.

The discussion concluded with recognition that prevention studies are needed in addition to treatment studies. Developing an efficient way to accurately and correctly identify MZ carriers early in their lives could provide a prevention approach for these individuals in terms of counseling and behavior modification,

*The Alpha-1 Foundation Therapeutic Development Network*

Stephen Rennard, MD, University of Nebraska Medical Center

There are several rationales for the Foundation’s Therapeutic Development Network (TDN). First, there are growing opportunities for innovation. Second, drug development is slowed or stalled because enrollment is often difficult and there is competition for innovation. Third, the patient community has little influence on development priorities and a network provides that opportunity. The TDN is currently in development. It will be a network of clinical trial sites with a dynamic patient database and a coordinating center. The coordinating center will assist in prioritizing and scheduling subject screening, facilitating data-driven protocol design, and enabling subject participation through multiple network sites. The Foundation will provide a voice in prioritizing the studies for innovation.

Opportunities for innovation are apparent from the discussions at this workshop and as demonstrated by multiple trials in Phase 1 through 3 development. Multiple therapeutic opportunities exist along the pathway of AATD pathogenesis, for example: correcting the abnormal gene, degrading the pathological mRNA, degrading the abnormal protein, blocking polymerization, repairing cirrhosis, replacing elastase inhibitors, and repairing emphysema. The TDN provides the infrastructure and resources to shorten study timelines and thereby lower the costs of drug development. It can provide a coordinating network for clinical trials, facilitating interactions and communications among sponsors, investigators, study sites, research participants, regulators, and payors. To date, the TDN protocol has been completed and received institutional review board approval. The data management system is in process and six beta sites are preparing to launch, with subject enrollment beginning in October 2023. Ultimately, another 22 sites will become network partners. The availability of so many sites will boost enrollment numbers and ease of access for research participants.

*Concluding Remarks*

Scott Santarella, Alpha-1 Foundation; Andrew Wilson, MD, Boston University

In summary, a growing body of evidence supports the concept that Z heterozygosity increases the risk for lung or liver disease in combination with additional environmental exposures or disease states. These insults are known to include tobacco smoking (lung disease) and obesity, diabetes, and alcohol consumption (liver disease). Despite this increasing understanding of risk, however, many significant details remain undefined. The population of Z heterozygotes is quite large in the United States and Europe. Given the hypothesized contribution of genetic cofactors to disease risk in this population, it is likely that risk is not uniformly distributed and instead might be higher among a proportion of the population. In addition, the mechanisms through which Z heterozygotes manifest disease and the extent to which these mechanisms mirror those in ZZ individuals remain unclear. Finally, whether treatment modalities currently in use for ZZs or under investigation in clinical trials or preclinical studies might confer a benefit to some Z heterozygotes remains unknown.

The sheer size of the MZ population provides a strong rationale to address these uncertainties even if only a small proportion are at significantly increased risk. As data emerge from ongoing trials of ZZ patients, we envision that it will inform our approach to addressing some of the questions outlined above. Focused investigation to identify disease mechanisms, determine genetic factors that influence disease susceptibility, and to identify subpopulations of MZ heterozygotes who are at highest risk are essential and will facilitate the ability to successfully execute clinical trials of existing and emerging therapeutics. The Alpha-1 Foundation is focused on supporting efforts to address these areas of uncertainty through a variety of means including the direct funding of research, continued investment in the Alpha-1 Registry, outreach to Alpha-1 patients, working with industry partners and regulators to establish acceptable endpoints for clinical trials, and working with industry partners to ensure that design of upcoming trials meets the needs of the patient community. Among those efforts, the TDN is being formed to facilitate the successful enrollment of clinical trials as they occur in the coming years.

## Data Availability

No original data were included in the manuscript. As a meeting proceedings, the manuscript is a summary of meeting presentations with relevant references included in the manuscript.
